# Employing Multiple Mini Interviews in Selection Processes for Psychology Professional Training Programs: Ten Tips for Effective Implementation

**DOI:** 10.15694/mep.2020.000192.1

**Published:** 2020-09-14

**Authors:** Melissa Oxlad, Rachel Roberts, Anna Chur-Hansen, Michael Proeve, Jaime Auton, Aspa Sarris, Alexandra Tabe

**Affiliations:** 1 University of Adelaide

**Keywords:** psychology, program selection, program admission, multiple mini interviews, interview processes

## Abstract

This article was migrated. The article was marked as recommended.

In Australia and internationally, entry into many professional psychology training programs is highly competitive, and as a duty of care to the public, training institutions must seek to offer places to those best-suited to training to become a psychologist. Typically, part of this selection process involves an interview. While panel interviews have been widely utilised, recently, in a range of health disciplines, these have been substituted for multiple mini interviews (MMIs) with evidence for their acceptability, validity and reliability. There is limited research on the use of MMIs in psychology professional training selection processes. We have used this approach for three years to select approximately 100 trainees for Clinical, Health and Organisational and Human Factors postgraduate psychology training programs. Based on our experience and feedback from applicants, we provide information that suggests this selection method is well-received by applicants. We also provide ten tips on how to effectively implement this approach to determine those most suitable for further training.

## Introduction

In Australia as well as internationally, entry into a professional training program in psychology leading to registration is an extremely competitive process with considerably more applicants each year than places. Therefore, significant responsibility is conferred on program staff to select the best candidates for training to become a psychologist ( Clark, Miller and Garwood, 2019). As a duty of care to the profession and the public, those selected must be deemed suited to enter the profession. One such means to assess suitability is through interviews.

As with other health professions, psychology is required to fulfil standards and expectations prescribed by an accreditation body (and in Australia, this is the Australian Psychology Accreditation Council). Accreditation bodies in psychology, as with medicine or other health professions, dictate minimum standards required for entry to training, and so any selection process must comply with these. Where selection for interviews is based on past academic performance in a sequence of undergraduate study in psychology as required for accreditation, along with referee reports and previous psychology-related employment or volunteering, the interview process can focus on applicants’ personal attributes. The personal qualities considered important in selection for training to become a psychologist are common to most health professions, and typically include interpersonal skills, critical thinking, ethical/moral judgement and receptiveness to feedback ( Callwood *et al.*, 2018; Lemay *et al.*, 2007; Reiter and Eva, 2005). Along with demonstrated knowledge about psychological practice and evidence of relevant experience, interviews aim to determine if applicants have the appropriate personal attributes that will allow them to be successful in the program as well as in their future careers within the profession.

Historically, many selection interviews for professional psychology degrees have adopted the panel format where the applicant answers questions and performs tasks, such as role-plays with several interviewers, who are typically members of faculty, professional psychologists and sometimes, may include members of the public. The use of such interview formats for selection into professional degrees in psychology, as well as other health professions, are open to multiple biases. Goho and Blackman (2006) summarise numerous issues with selection interviews in general including discriminatory biases (regarding characteristics like age or ethnicity), the halo effect (generalising from one rating on a characteristic to others), the rating bias of the interviewer (which can range from lenient to harsh, with some raters falling consistently in the centre), and biases related to the order in which applicants are interviewed (the contrast effect). For applicants, high stakes selection processes of any type may provoke performance anxiety. A panel interview may be especially stressful, particularly where the interviewer and applicant have a previous relationship (e.g., student and teacher/supervisor), where the applicant feels that they are underperforming during the interview or are being visibly rated during the process.

While no selection method has been developed to date which provides flawless predictive validity and affords minimal stress to applicants, Callwood *et al.* (2018) has argued that “universities have a duty to choose admissions processes that are as valid, defensible, and reliable as possible” (p. 56). As such, selection processes in many health professions have moved away from panel interviews to the more evidence-based and psychometrically robust Multiple Mini Interview (MMI) format ( Eva *et al.*, 2004). The MMI approach involves several brief interview stations. Typically, each station consists of one interviewer who asks each applicant a small number of standardised questions designed to assess personal attributes such as empathy, interpersonal skills and ethical values. As an admissions tool, the MMI focuses on the interpersonal capabilities of applicants and is used to assess their suitability for training ( Eva, Macala and Fleming, 2018; Eva *et al.*, 2004).

The structure of the MMI provides benefits in terms of increased efficiency allowing far more applicants to be interviewed in a short period ( Dodson *et al.*, 2009). Each applicant completes a pre-determined number of stations with a brief interval to transition from one station to the next. For each MMI station, time is allocated for interaction and assessing applicant performance and to complete an assessment form after the applicant has left the interview room. Additionally, the order in which applicants complete questions does not impact their performance on the MMI, which allows each interviewer to begin at the same time and for applicants to cycle through each station ( Kim and Kwon, 2018). The flexibility to complete stations in any order is highly desirable for practical reasons.

In addition to affording time and logistical benefits, MMIs attempt to mitigate various biases that can affect panel interviews. For example, greater standardisation can be achieved from one interviewer asking each applicant the same question. Ensuring that each applicant is asked the same set of questions and is rated by the same person on their answer enhances the reliability of MMIs over the traditional panel interview ( Bronwell, Lockyer and Lemay, 2007; Campagna‐Vaillancourt *et al.*, 2014; Dore *et al.*, 2010; Humphrey *et al.*, Goodyear, 2008; Kelly *et al.*, 2014). MMIs also allow assessors to be free from the bias that the influence of other interviewers may present, as they are scoring applicants individually ( Alweis, Fitzpatrick and Donato, 2015). Additionally, when compared to panel interviews, MMIs have been found to be superior in terms of reliability and predictive power ( Reiter *et al.*, 2007; Rosenfeld *et al*., 2008).

To date, MMIs have primarily been used as a selection tool in medical programs due to their reliability, validity, and efficiency ( Bronwell *et al.*, 2007; Campagna‐Vaillancourt *et al.*, 2014; Dore *et al.*, 2010; Griffin *et al.*, 2018; Humphrey *et al.*, 2008; Kelly *et al.*, 2014; Kumar *et al.*, 2009; Roberts *et al.*, 2014).

Implementation of MMIs has resulted in better predictions of performance in training programs ( Jerant *et al.*, 2018). Reports on the acceptability of MMIs have varied; some applicants have found them stressful, while others have described them as much more desirable than a traditional panel interview ( Boysen-Osborn *et al.*, 2018; O’Brien *et al.*, 2011; Soares *et al.*, 2015). These differences may relate to the nature of the program being applied for and the information available to applicants before the interview. The impact of applicant diversity factors on MMI performance has also been examined ( Clark, Miller and Garwood, 2019; Reiter and Eva, 2018). In their review, Reiter and Eva (2018) found variability in correlational findings across factors such as age, gender, cultural background, socioeconomic status, and rurality attributed to variations in MMI construction, content, and format, as well as differences in applicant pools and institutions. Other findings with regards to age bias are mixed, with some researchers reporting no differences in performance based on age ( O’Brien *et al*., 2011). Also, in some research, females have been reported to be advantaged when completing MMIs ( Ross *et al.*, 2017).

Given the strength of the MMI process in terms of psychometric properties and logistical advantages compared to the traditional panel interview, it is unsurprising that this form of interview has been implemented during selection processes within multiple healthcare professions (including medicine [ Patterson *et al.*, 2016], dentistry [ McAndrew and Ellis, 2012], nursing [ Perkins *et al.*, 2013], pharmacy [ Stowe *et al.*, 2014] and veterinary science ( Hecker *et al.*, 2009). However, there is a paucity of research examining the use of MMIs during the selection process for postgraduate psychology programs. Clarke and colleagues (2019) appear to be the first and only team of researchers that have investigated the feasibility and acceptability of MMIs during the selection into a postgraduate psychology program. Using a mixed-methods approach to data collection, Clarke *et al.* (2019) reported various applicant reactions to the MMI process, including factors associated with satisfaction and dissatisfaction.

While the findings of Clarke *et al.* (2019) are a useful start to understanding how the MMI process can be effectively implemented within the profession of psychology, there are a few limitations which question the generalisability of the findings. Specifically, the data was collected from a sample of applicants applying for entry into a graduate clinical psychology program within the United States of America (USA). Moreover, Clarke *et al.* (2019) included only one year of data on applicant satisfaction. It is unclear whether applicant reactions to the MMI process would be similar within universities outside of the USA, especially for those applying for entrance into other specialist psychology programs (such as health and organisational).

This study aimed to examine the feasibility and acceptability of the MMI process for candidates applying for entrance into one of three postgraduate psychology programs (Clinical, Health, and Organisational and Human Factors) at the University of Adelaide (Australia) across two separate years and intakes of students. While the School of Psychology has utilised MMIs for selection to its Master of Psychology programs since 2017, we sought feedback from applicants using exit interviews post-MMI in 2018 and 2019. We hoped to use this data to improve the acceptability and effectiveness of our MMI processes in the future. Based on our experience, in this article, we present ten tips for those considering the use of MMIs as part of their admission processes for professional psychology training programs.

## Perspectives of MMI interviewees

The University of Adelaide Human Research Ethics Committee approved this research (H-2018-18/86). In 2018 and 2019, after completing the MMI, in a room separate to the interviews, a member of the administrative team gave applicants an information sheet with a link to the online exit survey. Applicants were encouraged to complete the anonymous survey and were told that participation would not impact the outcome of the selection process. Across the two years, 119 applicants (91 females, 26 males, 1 other, 1 not-specified) provided feedback via the exit survey. Most applicants identified as Australian (53.8%) and 26.9% spoke a language other than English. Most applicants were interviewed for only one of the three programs (78.2%), and relatively few applicants had completed an MMI for selection into a postgraduate psychology program previously (20.2%) compared to those who had previously completed a panel interview for selection (43.7%).

Overall, the MMI process was well-received by applicants who appeared to appreciate the differences from a panel interview. One applicant summarised the process by saying, *‘I appreciate talking to someone one on one, rather than the traditional three on one panel. I did find it good to get up, take a break, change rooms. And in a small way I felt buoyed when I saw other interviewees in the corridor. It really was quite great, compared to other interviews.’* Other applicants expressed similar sentiments; *‘Facing only 1-2 people at a time was certainly less intimidating than being faced with a panel of 3 or more. You also feel like you have more of a chance of making a good impression. If you mess up one mini-interview then you start fresh with the next person’* and *‘Overall it was a positive experience and preferable to panel interviews.’*

Applicants further noted; *‘* *Although still stressful, especially not knowing what it would be, I found it much better than a panel’* and *‘Not feeling overawed by having to face a large panel of interviewers’.* The opportunity to be assessed by multiple interviewers was also appreciated by applicants who commented, *‘* *Getting to meet all new people and have your performance judged separately. Also made me less nervous when I was able to recover in between questions’,* *‘Felt less stressed because each session was short, so if one session didn’t go well I could start again in the next’*, and *‘Having different interviewers allowed me to answer confidently with the next interviewer if I fumbled the previous one.’* Additionally, having a smaller number of interviewers per station was seen as beneficial, *‘Relaxed set up and speaking with one person at a time made situation more comfortable.’*

To share what we have learnt from our experiences and the feedback from applicants, we present ten tips to assist other psychology programs who may want to implement MMIs as part of their admission processes when selecting candidates for professional psychology training programs.

## Tip 1: Stress Minimisation

### Providing clear and timely information about the application and interview process minimises applicant stress

While securing an interview for a highly sought after program with a limited number of places may always attract a certain level of stress, program staff can assist in minimising undue stress by ensuring that clear information about application and interview processes is made available to applicants in a timely manner. This information should be made available in course admissions guides, through university websites and promotional materials and the interview invitation. Having such information freely available is also an equity issue such that applicants that are not from the institution to which they are applying have access to the same information as those who have previously or are currently studying there. Applicant feedback supported this recommendation, for example, ‘ *Provide information re structure (MMI) prior to interview date especially for external/ interstate applicants who don’t have the advantage of knowing people who may have done it previously.’*

## Tip 2: Process Clarity

### Before the interview applicants must know they will undertake an MMI and what that entails

Given MMIs are relatively new to the field of psychology, and most applicants will not have completed one previously, it is essential that in course application guides and notification of offer for an interview, it is clear that the interview will be an MMI. This information must explain the MMI process, including preparation time, the number of stations, and the timing of and between stations. While applicants tended to respond positively to the MMIs, they did request that information about this format was made available before the interview. For example, *‘* *Perhaps pre-empt students it would be a Multiple Mini Interview:)’,* *‘* *give people a better understanding of what may be required of them in the interview. I had no idea what a mini interview was or what might be expected of me before the interview took place’,* and *‘Give some more info beforehand about what the process involves’.*

Applicants also requested information about the number and timing of stations and the overall interview length. For instance, *‘possibly provide more information about number and timing of stations’* and *‘Maybe prior information on how long each interview would be’.* We recommend, as suggested by one applicant, *‘It would be helpful to be emailed the outline - i.e., ethical scenario, two behavioural interview questions and a program specific interview.’*

## Tip 3: Question Selection Is Key

### Choose questions wisely to assess specific qualities and communicate the kinds of questions that will be asked to applicants

Program staff must give careful consideration to the qualities that they seek in trainee psychologists and must design their interview stations and questions to assess these qualities. While applicants will not be privy to the exact questions before presenting to the interview, information about the kinds of qualities required for a career as a registered psychologist and the types of questions that will be asked should be provided. Providing such information allows for transparency about the process and aids with applicant preparation. Applicants indicated that this would be useful in general, *‘Advise more ahead of time sort of questions will be asked to help preparation’*, but they particularly wanted advance notice that there would be a role play, which we had included as part of our MMI process: *‘Maybe give a heads up that there will be a role play’.*

## Tip 4: Preparation Aids Performance

### Provide questions in the holding room ahead of the interview

In our MMI process, administrative staff directed applicants to a holding room, on arrival, once their identity had been confirmed. Administrative staff acted as “marshals”, taking applicants from the holding room to their first station, facilitating timekeeping, leading applicants to a debriefing room after the MMI, and showing each applicant out of the building once their MMI process was complete.

Applicants should be provided with the questions that will be asked in each station immediately before their MMI. In the holding room, they should be allowed sufficient time to read the questions and to make notes in response to each question on the question paper provided. While making their notes, applicants must not refer to any pre-prepared notes as these should not be allowed in the holding room, nor should they have access to the Internet to research possible answers. Applicants should be allowed to take these notes and refer to them during their interview. All questions papers and notes must be returned to interview marshals after the interview; such documents must not leave the interview venue. Providing the questions in this manner, hopefully, helps to ease nerves and enables students time to consider their responses so they can maximise their interview performance. Applicants indicated that they valued this approach. For example, *‘Having the questions prior to the interview provided time for reflection to answer the questions effectively’* and *‘Taking notes beforehand was great.’*

## Tip 5: Timing Is Everything

### Ensure sufficient time for applicants to answer questions adequately at each station

When planning the timing of interviews, program staff need to ensure that each station has sufficient time to allow applicants to adequately answer the questions posed. Some applicants flagged they would have liked additional time, for example, *‘* *Allow a little more time for candidates to respond and not feel such strong time pressure, so candidates can take a moment if they need to think.’* Understanding the concept of MMIs, applicants only asked for stations to be slightly longer *‘Perhaps offer a little more time’.* When considering time, to maximise efficiency, it is also useful to structure the MMI such that each station runs for the same length of time.

## Tip 6: Fair Warning

### Provide applicants with an unobtrusive one-minute warningbefore the end of each station

As part of addressing concerns about timing and enabling applicants to adequately answer the questions posed at each station, it is important to have a mechanism to warn applicants that they only have one-minute remaining on the station. However, the method selected must seek to minimise distraction to applicants. During our MMI process, the one-minute warning was indicated by a knock on the interview station’s door. Unfortunately, this signal was perceived by many applicants as distracting with such comments as *‘* *Less distracting cue for one min remaining’* and *‘Less distracting one minute warning’.* Therefore, it is recommended that applicants are advised that they will receive a one-minute warning; this warning must be clear, yet unobtrusive (such as a bell or chime).

## Tip 7: Transition Takes Time

### Transitioning applicants between stations takes longer than you think

Time is also a critical consideration for the time allowed between stations. Program staff must give themselves ample time to move applicants between stations. We would recommend more time than you think you will need. Applicants indicated that they would have liked a little more time between stations so as not to feel rushed and to gather their thoughts. For instance, *‘A minute or so in between stations, so I can better collect my thoughts.’* and *‘Allowing time to have a bit of a breather after stations could help to calm nerves.’* As well as being advantageous to applicants, the time between stations also allows interviewers to finalise ratings and prepare for the next applicant.

## Tip 8: Experience Counts

### Utilise a pool of experienced interviewers with professional training in psychology

To aid success and complete the MMIs efficiently it is essential to have a pool of experienced registered psychologists to act as interviewers. It is beneficial to have a balance of academic registered psychologists and currently practising registered psychologists associated with the programs as affiliates of the University. There should be only one interviewer (or two in the case of role-play stations) per station to provide a less intimidating experience for applicants and to allow a wider number of interviewers to assess applicant suitability. While each interviewer rates the applicant on the questions specific to their station, it is recommended that all interviewers also assess applicants’ interpersonal skills.

## Tip 9: Uniformity Promotes Equity

### Equity is promoted when each station adheres to standardised processes

There must be a set of standardised processes for each station to ensure an equitable approach. An interview briefing must be held to familiarise each interviewer with their questions, the process for interacting with applicants, and how and when to score performance. Interviewers should be encouraged to adhere to the standardised approach but to also provide a safe and welcoming environment for applicants.

As part of standardisation, each station must follow the same process, including having a standardised beginning that explains the timing and builds rapport. Rather than immediately commencing with the first question, the interviewer should briefly introduce themselves and their role in the program. Some applicants noted that *‘Interviewers did not introduce themselves’*, that *‘Some panel members were stand offish’* and that it would be helpful to *‘Give a minute or two for introductions’* and *‘Brief small talk before asking questions.’* In contrast, applicants appreciated *‘friendly interviewers who made [them] feel comfortable and welcome’.*

It is also essential for interviewers to explain the time allocated for their station, as one applicant noted inconsistencies between interviewers which may lead to difficulties; *‘Some explained time allocation, some didn’t, I had no issues but some might have felt cheated of time.’* Finally, interviewers should not enter applicants scores until after the applicant has left the interview room. Where interviewers entered scores before the applicant had left the room, applicants indicated that *‘Viewing the interviewers mark out response whilst in the room’* was something that should be changed.

## Tip 10: Applicant Questions Matter

### Allow an opportunity for applicants to ask generic questions

As applicants are likely to have applied to multiple programs and are hoping to have a range of offers to choose from, they must also be able to ask generic questions about the process, the program and the institution. Many applicants indicated they had a preference to ask questions; ‘ *I had some questions to ask post interview and was not very happy that I didn’t get to.*’ and *‘* *We should have time to ask questions about the course’.* They suggested that the MMI process should *‘..allow time for interviewees to ask questions of their own’.* To cater for such questions, it is recommended that a member of academic staff and a student volunteer from within the professional training program be present in the debriefing room to answer generic questions.

## Conclusion

MMIs, while a relatively new experience for most applicants to psychology professional training programs, are well received and acceptable to applicants. Indeed, in our experience, many applicants reported a preference for MMIs compared to traditional panel interviews. Given this level of acceptability, together with MMIs enabling program staff to interview a larger volume of applicants in order to select those most suited to being offered a place in highly competitive professional training programs, and their ability to address many of the biases inherent in panel interviews, MMIs appear to offer a valuable opportunity for selection processes. The tips provided will enable psychology programs to implement MMIs effectively. We have provided a user-friendly checklist to aid in the planning and implementation of MMIs (see Table 1).

**Table 1.** Checklist to prepare for undertaking MMIs

Tasks	If Yes	If No
Have you designed clear application and interview information?	Proceed to next step	• Speak with your institution’s Admissions staff and work together to devise appropriate marketing materials.
Have you ensured application and basic interview information is publicly available to potential applicants in a timely manner?	Proceed to next step	• Arrange for your institution’s Admissions and Marketing staff to disseminate the course information widely - hard copies, websites and social media, video, careers sessions before applications open.
Have you considered the qualities that you wish to select for and devised questions that will assess these qualities?	Proceed to next step	• Work with colleagues to agree on qualities that you wish to assess for each program. • Devise questions which will assess these qualities.
Have you decided the number of stations that will be required?	Proceed to next step	• Work with colleagues to determine how many stations are needed to assess generic qualities and suitability for specific programs.
Have you ensured that each station will be the same length?	Proceed to next step	• Review the time needed for each station. • • If necessary, add or remove questions from some stations to ensure each station can complete all questions in the same amount of time.
Have you decided how much time you need to transition between stations? Have you tested your timings?	Proceed to next step	• Discuss timing with colleagues and with others who have experience with MMIs. • Consider adding additional time to the first figure chosen as typically more time is needed than allocated.
When advising applicants they have been offered an interview, will the notification clearly explain that they will participate in an MMI and what that entails?	Proceed to next step	• Work with your institution’s Admissions staff to generate an email and/or letter that details this information.
Will applicants be made aware of the kinds of questions that will be asked at interview?	Proceed to next step	• Work with your institution’s Admissions staff to generate an email and/or letter that details this information. • This should be provided at the time the offer of interview is made.
Have you decided how will you provide applicants with a one-minute warning?	Proceed to next step	• Discuss different methods with colleagues and with others who have experience with MMIs. • Select least disruptive method and ensure applicants are advised of this before their stations commence.
Have you decided how much time applicants will be given in the holding room and considered additional time for those being interviewed for more than one program?	Proceed to next step	• Determine times for those completing one interview versus those being interviewed for more than one program. • Consider scheduling different interview times for those being interviewed for one program versus those being interviewed for more than one program to make holding room timing more efficient.
Have you gathered a pool of suitably qualified and experienced interviewers?	Proceed to next step	• Discuss with colleagues. • Contact other registered psychologists affiliated with your institution to determine their willingness and availability to assist.
Have you gathered enough Marshalls to assist during MMIs?	Proceed to next step	• Speak with your institution’s Admissions staff to ensure there are sufficient Marshalls.
Have you developed a standardised approach for the stations?	Proceed to next step	• Discuss with colleagues. • Write up standardised process to aid communication to all parties including interviewers and Marshalls.
Have you communicated all standardised requirements to interviewers?	Proceed to next step	• Ensure this is done prior to interviews. • This is often best delivered verbally in a briefing prior to the commencement of each block of interviews.
Do applicants have an opportunity to ask questions?	Well done!	• Determine the optimal time for applicants to ask generic questions such as in the debriefing room after all stations have been completed. • Determine who will be present to answer questions - Staff? Students currently in the programs?

## Take Home Messages

MMIs are an effective interview method for selecting candidates for professional training in psychology. To effectively implement MMIs, ensure:

• Information about MMIs are included in all application and interview information disseminated to applicants and that applicants are provided details about what the MMI will entail.
• The timings for the MMIs are carefully chosen to ensure enough time for applicants to answer questions adequately and to move between stations with ease.
• A pool of experienced interviewers is available and that all stations follow a standardised approach.

## Notes On Contributors

**Melissa Oxlad** is a Lecturer, School of Psychology, The University of Adelaide, Australia. She is a registered psychologist, Co-Program Director of the Master of Psychology (Health) and Academic Lead for Internships and Employability. ORCID ID: https://orcid.org/0000-0001-9067-6706

**Rachel Roberts** is an Associate Professor, School of Psychology, The University of Adelaide, Australia. She is a registered psychologist, the Head of the School of Psychology and Academic Lead for Master Program Admissions. ORCID ID: https://orcid.org/0000-0002-9547-9995

**Anna Chur-Hansen** is a Professor, School of Psychology, The University of Adelaide, Australia. She is a registered psychologist and Co-Program Director of the Master of Psychology (Health). ORCID ID: https://orcid.org/0000-0002-2935-2689

**Michael Proeve** is a Senior Lecturer, School of Psychology, The University of Adelaide, Australia. He is a registered psychologist and Program Director of the Master of Psychology (Clinical). ORCID ID: https://orcid.org/0000-0002-4710-585X

**Jaime Auton** is a Lecturer, School of Psychology, The University of Adelaide, Australia. She is a registered psychologist and Course Coordinator for courses in the Master of Psychology (Organisational and Human Factors). ORCID ID: https://orcid.org/0000-0002-4843-5519

**Aspa Sarris** is a Senior Lecturer, School of Psychology, The University of Adelaide, Australia. She is Program Director of the Master of Psychology (Organisational and Human Factors). ORCID ID: https://orcid.org/0000-0001-6819-8883

**Alexandra Tabe** is a Psychology Student and Research Assistant, School of Psychology, The University of Adelaide, Australia.
