# Supplementary material for: Employing Multiple Mini Interviews in Selection Processes for Psychology Professional Training Programs: Ten Tips for Effective Implementation
Source: MedEdPublish (2016). 2020 Sep 14;9:192. [Version 1] doi: 10.15694/mep.2020.000192.1 (PMC10699392; doi:10.15694/mep.2020.000192.1)
Supplement: Peer review history

# Reviewer report: Reviewer response for version 1

**DOI:** 10.21956/mep.20140.r31103

**Published:** 2020-11-28

**Authors:** Ken Masters

**Affiliations:** 1 Sultan Qaboos University

This review has been migrated. The reviewer awarded 4 stars out of 5 A useful Tips paper on using multiple mini interviews (MMIs) in selection processes for psychology professional training programs.The basis for the tips is strong, relying both on literature and the authors’ own research. Although the context is psychology, much of the contents could be more generally applicable, especially if the institution is new to MMIs.The checklist is also a useful tool.My only real criticism of the paper lies in the Abstract and the Take Home Message:o The Abstract gives good background information, but would be more useful if it gave a summary of the tips (in much the same way that a research paper gives a summary of the results). (A focus of the paper is on the tips, yet they are mentioned in only short one sentence in the Abstract).o In its brevity, the Take Home Message tends to sell the value of the paper short.Overall, however, an interesting and valuable read.

Reviewer Expertise:

NA

No decision status is available

# Reviewer report: Reviewer response for version 1

**DOI:** 10.21956/mep.20140.r31102

**Published:** 2020-11-11

**Authors:** Barbara Jennings

**Affiliations:** 1 Norwich Medical School

This review has been migrated. The reviewer awarded 4 stars out of 5 This article presents guidance on the use of multiple mini interviews (MMIs) for selection of course applicants. The authors are discussing the recruitment for a postgraduate psychology programme, but I think it will resonate with and be of great interest to admissions tutors for many undergraduate or postgraduate clinical schools.In their introduction, the authors cite useful background literature and present the rationale for using MMIs in preference to a traditional panel format because of evidence base and in particular for psychometric robustness. I found the exploration of bias particularly thought provoking.The tips outlined in the main body of the article are based on faculty experience and the analysis of feedback from exit interviews (after applicants had completed their MMI). There is detailed and wise advice around timings of the interview steps and a very helpful checklist for planning an interview protocol.There is much to recommend in this article. It is well researched, clear and well presented and the authors describe governance steps linked to the applicants’ feedback and data presented.

Reviewer Expertise:

NA

No decision status is available
